# Phytochemical and Pharmacological Insights into *Mentha asiatica* Boriss.: A Promising Antimicrobial Plant

**DOI:** 10.3390/molecules30030511

**Published:** 2025-01-23

**Authors:** Baiken Baimakhanova, Amankeldi Sadanov, Gul Baimakhanova, Diana Tluebekova, Alma Amangeldi, Zere Turlybaeva, Irina Ratnikova, Zhanar Nurgaliyeva, Roza Seisebayeva, Botagoz Ussipbek, Lyazzat Umbetyarova, Akerke Amirkhanova, Gulnaz Seitimova, Aknur Turgumbayeva

**Affiliations:** 1 Research and Production Center for Microbiology and Virology LLP, Bogenbay Batyr St. 105, Almaty 050010, Kazakhstan; bbbayken@mail.ru (B.B.); a.sadanov1951@gmail.com (A.S.); bgulb@mail.ru (G.B.); dianatleubekova1@gmail.com (D.T.); almashka91@mail.ru (A.A.); tzj2009@yandex.kz (Z.T.); iratnikova@list.ru (I.R.); 2 School of Pediatrics, Department of Outpatient Pediatrics. S.D. Asfendiyarov Kazakh National Medical University, Almaty 050010, Kazakhstan; nurgaliyeva.z@kaznmu.kz (Z.N.); seisebaeva_68@nail.ru (R.S.); 3 Department of Biophysics, Biomedicine and Neuroscience, Al-Farabi Kazakh National University, Almaty 050040, Kazakhstan; 119bota@gmail.com (B.U.); umbetyarovalyazzat75@gmail.com (L.U.); 4 School of Pharmacy, S.D. Asfendiyarov Kazakh National Medical University, Tole-bi 94, Almaty 050012, Kazakhstan; 5 Faculty of Chemistry and Chemical Technology, Al-Farabi Kazakh National University, Almaty 050040, Kazakhstan; gulnaz.seitimova@gmail.com; 6 Higher School of Medicine, Al-Farabi Kazakh National University, Almaty 050040, Kazakhstan

**Keywords:** Asian mint, *Mentha asiatica* Boriss., extraction, essential oils, GC–MS analysis, phytochemical composition, antimicrobial activity, bioactive compounds

## Abstract

*Mentha asiatica* Boriss., a species native to Central Asia, has garnered significant attention for its diverse phytochemical profile and antimicrobial potential. This review synthesizes current knowledge on the antimicrobial activities of *M. asiatica*, focusing on its essential oils and other bioactive constituents. The study contextualizes the importance of natural antimicrobials in the era of rising antibiotic resistance and highlights the plant’s traditional use in ethnomedicine. The main methodologies explored include gas chromatography–mass spectrometry (GC–MS) for phytochemical characterization and various in vitro assays to assess antimicrobial efficacy against bacterial and fungal pathogens. The essential oils of *M. asiatica* demonstrate a broad spectrum of activity, attributed to compounds such as menthol, menthone, and carvone. Other phytochemicals, including sesquiterpenes and terpenoids, also contribute to its bioactivity. The review underscores the potential of *M. asiatica* as a source of novel antimicrobial agents and calls for further research into its mechanisms of action, bioavailability, and safety profiles. The findings position *M. asiatica* as a promising candidate for developing plant-based antimicrobial formulations, addressing critical needs in healthcare and food preservation.

## 1. Introduction

Ethnomedicine, the study of traditional medicinal practices, offers valuable insights into the therapeutic potential of plants [1,2,3,4]. Phytochemicals, the bioactive compounds within these plants, have shown significant pharmacological promise. Investigating these compounds, guided by ethnomedicinal knowledge, can lead to the development of novel pharmaceuticals while also contributing to the preservation of traditional knowledge and biodiversity [5,6,7].

**Figure 1:**
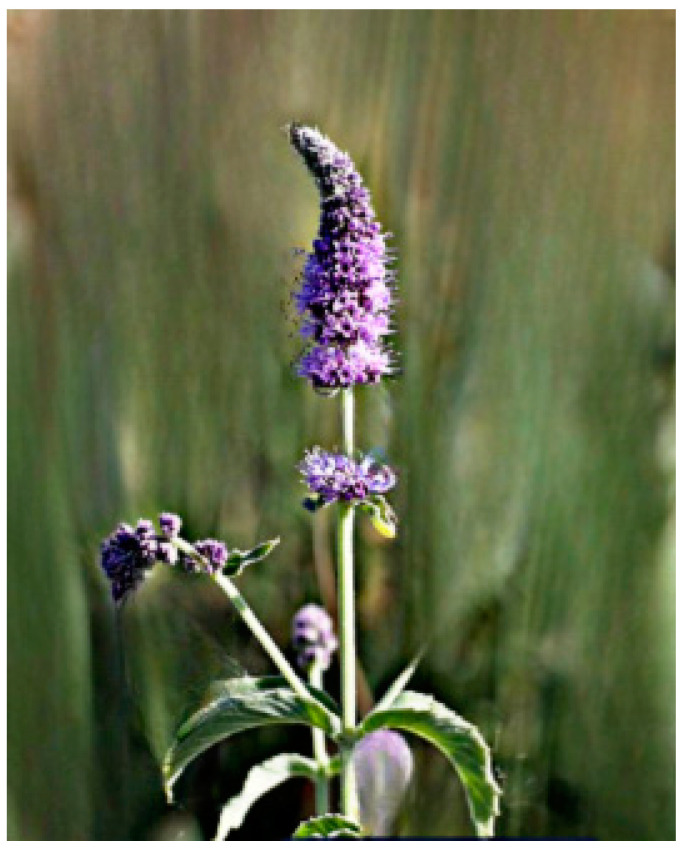
Asian mint (*M. asiatica* Boriss.).

The Lamiaceae family, comprising approximately 250 genera and 7,000 species globally, plays a vital role in both ecosystems and human cultures, thriving in diverse climates ranging from tropical to temperate regions [8,9]. *Mentha asiatica* Boriss. (Figure 1), a member of this family, is native to Asia and found in countries such as Kazakhstan, China, and India [10]. Also known as Asian mint, *M. asiatica* Boriss. is increasingly recognized for its medicinal properties, with traditional ethnomedicinal uses for its antioxidant, anti-inflammatory, antimicrobial, and analgesic effects [11]. The plant’s therapeutic potential stems from a variety of bioactive phytochemicals, including flavonoids, terpenes, and phenolic acids [12,13,14]. Furthermore, *M. asiatica* Boriss. has demonstrated antioxidant, anti-inflammatory, and antibacterial activities in research, making it a subject of growing interest in pharmaceutical development [15,16,17,18]. Its adaptability to diverse climates and wide distribution underscore its value as a genetic resource for medicinal purposes. Continued research and conservation efforts are crucial to fully unlocking the therapeutic potential of *M. asiatica* Boriss., offering new insights into its medicinal applications while supporting biodiversity conservation.

Plants have been essential for human survival, providing sustenance, shelter, clothing, and medicine. Their importance is particularly evident in less developed regions, where traditional medicine remains the primary healthcare system for an estimated 80% of the population [19,20,21,22]. Medicinal plants, including *M. asiatica* Boriss., are crucial in these systems, with over 3.3 billion people relying on them for regular healthcare [23]. Asian mint has a long history of use in traditional medicine, especially in Asia, valued for its antioxidant, anti-inflammatory, antimicrobial, and analgesic properties. Traditionally, it has been used to combat oxidative stress and inflammation, with its leaves, rich in terpenes and phenolics, employed to treat inflammatory conditions like arthritis. *M. asiatica* is also known for its antimicrobial activity, used against infections such as colds, coughs, respiratory ailments, and digestive issues like dysentery and diarrhea, demonstrating efficacy against both bacterial and fungal pathogens. Its analgesic properties have been utilized to alleviate pain from headaches, toothaches, and muscle aches, attributed to its ability to modulate pain perception and reduce inflammation [24,25]. Additionally, Asian mint is commonly used to support digestive health, acting as a mild stimulant for appetite and digestion, often consumed as teas or infusions to relieve bloating, indigestion, and nausea.

The study of phytoconstituents, including essential oils, in *M. asiatica* Boriss. is crucial for pharmacological research, given the plant’s rich array of compounds with diverse beneficial effects. These natural compounds hold significant pharmacological potential, making *M. asiatica* Boriss. a promising candidate for new therapeutic development. Understanding the pharmacological potential of these phytochemicals could lead to the creation of novel medicines for a wide range of diseases. This review aims to provide a comprehensive overview of the botanical characteristics, geographical distribution, phytochemical composition, and pharmacological properties of *M. asiatica* Boriss., promoting further scientific exploration and highlighting its potential health benefits.

## 2. Methods

A comprehensive literature search was conducted across scholarly databases, including Google Scholar and PubMed, using key words such as “*Mentha asiatica* Boriss.”, “*Mentha asiatica* Boriss. compounds”, “*Mentha asiatica* Boriss. phytochemicals”, “*Mentha asiatica* Boriss. pharmacological”, and “*Mentha asiatica* Boriss. traditional uses”. The criteria for article selection encompassed investigations into the traditional applications, phytochemical constituents, and pharmacological characteristics of the *M. asiatica* Boriss. genus. Additional relevant studies were identified through a review of the references cited within the selected articles. Relevant texts containing taxonomic and ethnobotanical information were also consulted. The data collection period spanned from 1980 to 2024.

## 3. Distribution and Botanical Characterization

**Figure 2:**
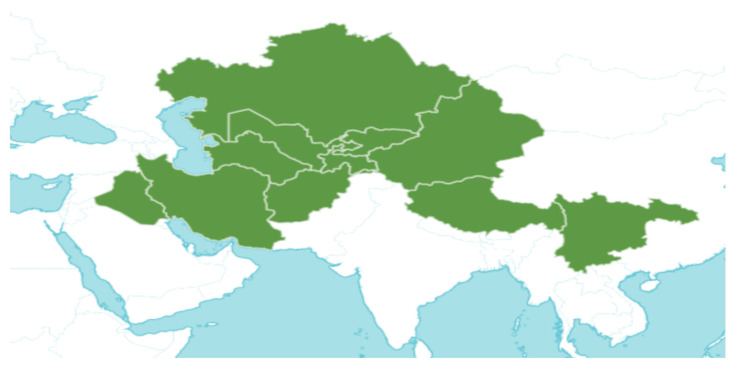
The distribution of *M. asiatica* Boriss. using shaded green color.

*Mentha asiatica* Boriss. belongs to the Lamiaceae family, comprising approximately 250 genera and over 7000 species globally. The wide distribution and diverse characteristics of the Lamiaceae family highlight its ecological and cultural importance, as its members contribute to biodiversity, traditional medicine, and culinary applications [11]. The genus *Mentha* L., within this family, includes about 25 to 30 species. Among them is *M. asiatica* Boriss., native to Afghanistan, South-Central China, Iran, Iraq, Kazakhstan, Kyrgyzstan, Tajikistan, Tibet, Turkmenistan, Uzbekistan, and Xinjiang (Figure 2). *M. asiatica* Boriss. thrives in temperate biomes, where it plays a significant role in ecosystems and holds the potential for pharmacological applications. Further research and conservation efforts are crucial to fully understanding the benefits of plants like *M. asiatica* Boriss. and preserving their biodiversity for future generations [22].

*M. asiatica* Boriss. is a perennial plant, typically reaching a height of 30–80 cm. Its stem is erect, quadrangular, and covered with fine pubescent hairs, which may vary in density along the stem. The ovate–lanceolate leaves, approximately 25–60 mm long and 10–30 mm wide, have serrated margins and an acute apex. They are arranged oppositely on the stem and are covered with fine glandular hairs that secrete essential oils. The flowers are arranged in verticillate clusters (whorls) located in the axils of the upper leaves. The inflorescence is compact and bracteate, with bracts that are smaller than the leaves and sometimes tinged with purple [23].

The calyx is tubular, 3–5 mm long, and covered with glandular and non-glandular hairs, with five triangular lobes. The corolla is light purple to lavender, measuring about 4–6 mm in length, with a tubular base and slightly spreading lobes. The upper lobe is slightly larger than the lower ones, with a rounded and slightly notched appearance. The four stamens are didynamous (two pairs of unequal length) and included within the corolla tube. The filiform style is 6–8 mm long, with a bifid stigma. The fruit is a schizocarp, splitting into four ovoid, smooth, brown nutlets, each 1.5–2 mm long. The plant has a strong aromatic fragrance due to its high essential oil content [26]. 

## 4. Technology for Obtaining Extracts from *M. asiatica* Boriss. Leaves

Extraction is the initial step in isolating bioactive compounds from natural sources. Common extraction methods include solvent extraction, distillation, pressing, and sublimation, each based on specific extraction principles. Solvent extraction is the most widely used technique [7]. The extraction process involves several sequential steps: solvent penetration into the solid matrix, solute dissolution in the solvent, solute diffusion out of the solid matrix, and collection of the extracted solution. Factors that enhance diffusivity and solubility in these steps improve extraction efficiency. These factors include the nature of the extraction solvent, particle size of the raw material, solvent-to-solid ratio, extraction temperature, and extraction time [27].

*M. asiatica* Boriss. is processed to obtain extracts using various extraction techniques, with hexane being a commonly employed solvent. A study using soaking extraction for rapeseed highlighted the principles of effective solvent interaction and component release, analogous to essential oil extraction from plants like *M. asiatica*. Water distillation, a traditional and efficient method for essential oil extraction, was used to isolate volatile compounds from *M. asiatica*. Gas chromatography–mass spectrometry (GC–MS) analysis of the extracted oil revealed a complex phytochemical profile. Thirty-seven compounds, representing 97% of the total components, were identified, with *trans*-piperitone oxide (64.51%) and piperitenone oxide (12.34%) as the major constituents [28]. This approach emphasizes the importance of optimizing extraction technologies and conditions, such as solvent choice, temperature, and duration, to maximize yield and purity. This highlights the potential of applying tailored methods to harness bioactive compounds from various plant species. Such studies focus not only on leaf extracts but also on the use of water distillation for extracting volatile oils from flowers, stems, and roots, where bioactive compound composition can vary significantly.

The volatile constituents of *M. asiatica* Boriss. were extracted through steam distillation, a method commonly employed for isolating essential oils due to its efficiency in preserving heat-sensitive compounds. The extracted oils were analyzed using GC and GC–MS, enabling precise identification and quantification of their chemical profiles. Leaf oil was dominated by piperitone (67.6%), with significant amounts of isomenthone (6.6%) and cis-piperitol (4.2%), whereas flower oil exhibited a distinct profile, primarily comprising piperitone (55.7%), carvone (16.2%), and pulegone (4.1%). These findings illustrate the impact of plant part specificity on the chemical composition of essential oils and emphasize the importance of using advanced analytical techniques like GC and GC–MS to characterize and compare these variations. Such studies guide the selection of appropriate distillation techniques and plant materials for targeted applications in pharmaceuticals, aromatherapy, and other industries. This method is also applicable for roots and seeds, where the chemical profile might indicate the presence of rare sesquiterpenes and fatty acid derivatives. Such insights expand the potential applications of steam distillation (SD) across various industries [29].

In another study, the aroma composition of *M. asiatica* Boriss. was investigated using the purge-and-trap extraction (PTE) technique, which efficiently isolates volatile organic compounds by capturing them in a cold trap and subsequently extracting them with dichloromethane. GC–MS analysis identified 33 compounds, including acids, alcohols, aldehydes, esters, hydrocarbons, and terpenes. Terpenes emerged as the predominant chemical group, with linalool being identified as the main terpene in *M. asiatica*. Among the esters, linalyl acetate stood out as a significant constituent, showcasing its role in the aromatic profile. This advanced combination of extraction and analytical techniques highlights the complex chemical composition of *M. asiatica*, underscoring its potential for applications in flavoring, perfumery, and therapeutics. Moreover, studies extending the PTE to other anatomical parts of *Mentha* species, such as stems and flowers, have identified broader spectra of esters and terpenoids, enhancing their applicability in cosmetics and aromatherapy [30].

Aimila A. et al. compared three extraction methods for volatile components of *M. asiatica* Boriss. These methods included lipophilic solvent extraction with *n*-hexane, steam distillation (SD), and supercritical carbon dioxide (SC-CO_2_) fluid extraction. Among the methods, n-hexane extraction produced the highest yield (1.27 ± 0.03%, w/W), followed by SC–CO2 (1.15 ± 0.04%, w/W), while SD yielded 0.37 ± 0.01% (w/W). Using gas chromatography quadrupole time-of-flight mass spectrometry (GC–QTOF–MS) and a gas chromatography–flame ionization detector (GC–FID), the study identified 70 compounds in the aromatic oil extract, with prominent constituents including *α*-thujene, camphene, sabinene, *β*-pinene, *β*-cymene, limonene, camphol, carvone, piperitone oxide, and caryophyllene oxide. These methods produced oils with significant antioxidant activity, as evidenced by their performance in the 2,2′-Azinobis-(3-ethylbenzothiazoline-6-sulfonic acid) (ABTS) assay and the [2,2-di(4-tert-octylphenyl)-1-picrylhydrazyl] (DPPH) assay. The oils also exhibited inhibitory effects against *Staphylococcus aureus*, *Candida albicans*, *Bacillus subtilis*, and *Escherichia coli*. Optimization of the SD method using response surface methodology (RSM) revealed that soaking time had the greatest influence on yield, followed by the solid-to-liquid ratio and extraction time, with optimal conditions achieving a 0.38% yield. This study highlights the influence of extraction parameters and techniques on the chemical and biological profiles of *M. asiatica Boriss.* essential oils. The versatility of SC-CO_2_ extraction has been further demonstrated in studies focusing on roots and seeds, yielding compounds like sterols and fatty acids with bioactive potential [31]. 

Moreover, Aimila A. et al. studied the chemical composition of essential oils extracted from the aerial parts of *M. asiatica Boriss.* in Xinjiang to understand its bioactive potential. The essential oil was initially separated using silica gel column chromatography and fractionated based on thin-layer chromatography results, yielding eight fractions. Each fraction exhibited varying levels of antibacterial activity, which was further investigated using preparative gas chromatography (prep-GC) for isolation. Ten compounds were identified using advanced techniques, including high-resolution ¹H and ¹³C Nuclear Magnetic Resonance (NMR), as well as GC–QTOF–MS. Key constituents included sabinene, limonene, *β*-caryophyllene, piperitone oxide, thymol, and 4-hydroxypiperidone. Notably, bioautography screening highlighted 4-hydroxypiperidone and thymol for their potent antibacterial activity, particularly against *Candida albicans*. These compounds significantly inhibited ergosterol synthesis in the fungal cell membrane in a dose-dependent manner. This study demonstrates the potential of *M. asiatica Boriss.* as a source of bioactive compounds and provides a foundation for its development into medicinal and pharmaceutical applications. The study of aerial parts such as stems and flowers has further enriched our understanding of the antibacterial potential of *Mentha* species, pointing to applications in antimicrobial coatings and pharmaceuticals [32].

In an additional study, the essential oil of *M. asiatica Boriss.* of Mongolian origin was analyzed using GC and GC–MS to identify its volatile components. The oil, extracted from plants collected in their natural habitat, was characterized by a high concentration of rosefuran oxide (63.17%) and rosefuran (11.56%), indicating a unique chemotype for *M. asiatica*. The extraction method ensured the preservation of these heat-sensitive compounds, with advanced analytical techniques like GC–MS and NMR spectroscopy confirming their identities [33].

In an additional study by Gazizova et al., the phytochemical composition and antioxidant properties of *M. asiatica Boriss.* leaves were thoroughly examined using various extraction methods, including Soxhlet apparatus ethanol extraction, vortex-assisted ethanol extraction, ultrasound-assisted ethanol extraction, and water extraction via hydrodistillation. The study revealed that piperitenone oxide was the predominant compound across all extraction methods, with concentrations ranging from 49.29% to 87.65%. Additionally, compounds such as alkanes, alkenes, ketones, terpenes, and fatty acids were identified, all known for their antimicrobial and antioxidant properties. The extract obtained using the ultrasound-assisted extraction (UAE) method exhibited the highest antioxidant activity, as evaluated by DPPH and ABTS assays. It also demonstrated significant antimicrobial effects, with remarkably low minimum inhibitory concentrations against *Candida albicans* and *Pseudomonas aeruginosa*, highlighting its potential for therapeutic applications [34].

The investigation into the extraction of bioactive compounds from *Mentha asiatica* Boriss. has been conducted utilizing a variety of methodologies, each aimed at optimizing distinct parameters to enhance yield while simultaneously preserving volatile constituents (Table 1).

Various advanced techniques have been developed for obtaining extracts from *M. asiatica*, each offering unique advantages based on the target compounds and desired applications. These methods are instrumental in isolating essential oils, bioactive compounds, and other valuable phytochemicals. For instance, water distillation and steam distillation are traditional methods widely used for extracting essential oils from plant materials. Water distillation involves immersing the plant material in boiling water, where steam carries volatile compounds into a condensation chamber. In contrast, steam distillation applies steam directly to the plant material, minimizing compound degradation. Studies on *M. asiatica* demonstrated the efficacy of these methods in preserving heat-sensitive compounds like piperitone and isomenthone, which are crucial for pharmaceutical and aromatherapy applications. The primary advantage of these methods is their simplicity and cost-effectiveness, making them suitable for large-scale production. However, the optimization of parameters like steam pressure and distillation duration is essential for maximizing yield and maintaining compound integrity [28,35].

The PTE technique is a sophisticated method designed to isolate volatile organic compounds by purging them from a sample matrix with an inert gas and trapping them in a cold trap. For *M. asiatica*, this technique efficiently captured terpenes, esters, and aldehydes, enabling the precise profiling of aromatic compounds using GC–MS. The advantage of PTE lies in its ability to preserve the delicate chemical composition of volatile compounds, making it ideal for studying the flavor and fragrance profiles of essential oils. Studies have highlighted its application in isolating linalool and linalyl acetate, compounds known for their therapeutic potential in stress relief and perfumery [29,36,37,38]. 

Lipophilic solvent extraction, using solvents like n-hexane, and supercritical CO_2_ extraction are highly effective for obtaining non-polar bioactive compounds. SC–CO_2_, in particular, offers a green and sustainable approach, leveraging the tunable solubility of CO_2_ under varying temperature and pressure. Research on *M. asiatica* revealed that these methods yield high concentrations of terpenes, alkenes, and fatty acids, with significant antioxidant and antimicrobial properties. The soxhlet apparatus method, while more time-intensive, ensures the exhaustive extraction of compounds, making it suitable for comprehensive phytochemical studies. These methods are advantageous for their high yields, reproducibility, and ability to target specific compound groups [31,34,39].

UAE employs high-frequency sound waves to create cavitation, disrupting plant cell walls and enhancing solvent penetration. This technique has been shown to produce high yields of piperitenone oxide and other antioxidants from *M. asiatica*, with reduced solvent and energy consumption. Similarly, VAE uses mechanical stirring to accelerate compound diffusion, offering a simple and rapid alternative for extracting bioactive compounds. Both UAE and VAE have been lauded for their eco-friendly nature, reduced extraction times, and compatibility with heat-sensitive compounds [34].

Silica gel column chromatography and preparative gas chromatography are advanced techniques used for fractionating and isolating specific bioactive compounds from complex extracts. For *M. asiatica*, these methods have successfully separated compounds like sabinene, thymol, and 4-hydroxypiperidone, which exhibit potent antibacterial and antifungal activities [30,32].

## 5. Phytochemistry

The phytoconstituents of *M. asiatica* Boriss. are diverse and exhibit a wide range of pharmacological activities, making the plant a valuable source for potential therapeutic applications [35,36,37,38,39,40,41,42,43,44,45,46,47,48,49,50,51,52,53,54,55,56,57,58,59,60,61,62,63,64,65,66,67,68,69,70,71,72,73,74,75,76,77,78,79,80,81,82,83,84,85,86,87,88,89,90]. For instance, the essential oils of *M. asiatica* Boriss. contain various bioactive compounds, including essential oils, terpenes and derivatives, saturated and unsaturated hydrocarbons, alcohols, fatty acids, phenolics, and other classes of compounds [30,31,32,33,34]. Notably, piperitenone oxide, a major compound in essential oils, has demonstrated significant antioxidant, antimicrobial, and anti-inflammatory properties [36,88]. Overall, the chemical classes identified in *M. asiatica* Boriss. include alkanes, alkenes, alkynes, alcohols, aromatic hydrocarbons, aldehydes, aziridine derivatives, bicyclic ketones, brominated alcohols, bicyclic compounds, chlorinated aldehydes, cycloalkanes, cyclic dienes, cyclic dioxanes, cyclic ethers, dihydrobenzopyrans, ethers, fatty acids, fatty acid methyl esters, fatty alcohols, unsaturated aldehydes, fatty amines, furans, fluorinated compounds, furan derivatives, heterocyclic compounds, ketones, lactones, phenolic compounds, sterols and derivatives, sulfonyl chlorides, spiro compounds, terpenes and derivatives, sesquiterpenes and derivatives, terpenoids, terpenoid esters, terpenoid alcohols, thiepenes, thienopyridines, thiophenes, organosilicon compounds, pyrazolone derivatives, and phenylpropanoids (Table 2).

*M. asiatica* is a valuable medicinal plant known for its diverse phytochemical profile, which includes a rich array of phenolic compounds and flavonoids. For instance, thymol and carvacrol, in particular, are well-documented for their biological activities. Thymol has shown strong antibacterial effects, particularly against Gram-positive bacteria, and is a promising candidate for use in antimicrobial formulations. Similarly, carvacrol has been studied for its ability to disrupt bacterial cell membranes, enhance oxidative stress in pathogens, and inhibit biofilm formation. Both compounds have also demonstrated antioxidant activity by scavenging free radicals and reducing oxidative damage, which are essential in preventing chronic diseases such as cancer and cardiovascular disorders. However, among the phenolic compounds isolated from this plant, such as phenol, 2-(2-propenyl), phenol, 5-methyl-2-(1-methylethyl), and phenol, 2-methyl-5-(1-methylethyl), their biological activities remain unexplored, and it is not yet known whether these compounds possess antimicrobial, antioxidant, or other properties [30,81].

According to the literature, flavonoid compounds have not specifically been studied in *M. asiatica*, highlighting a significant gap in understanding the phytochemical diversity of this species. To address this, advanced extraction techniques should be employed to efficiently isolate flavonoids and derivatives besides UAE, VAE, and SC–CO_2_ methods.

The chemical composition of *M. asiatica* Boriss. leaves is rich in diverse phytochemical classes, which are primarily responsible for its bioactivity. Identified compounds include alkanes such as undecane [34], dodecane [34], and tetradecane [34], which possess antibacterial, anti-inflammatory, anticancer, antifungal, and antioxidant properties [59]. Additionally, alkenes like 1-eicosene [34] and 1-tetradecene [34] contribute antimicrobial and anticancer activities, while alkynes such as 1-octen-3-yne [34] have antimicrobial properties [72]. Alcohols, including cyclohexanol [34] and bicyclo[2.2.1]heptan-2-ol [34], enhance the plant’s antifungal and antimicrobial profile [73]. Aromatic hydrocarbons such as benzene [34] and trimethylbenzene [34] offer antimicrobial and antioxidant effects [75,76]. Aldehydes like 2-hexenal [34] and 2-decenal [34] are also antimicrobial [77,78]. The plant contains sesquiterpenes and derivatives such as trans-caryophyllene [30,32,34] (anti-inflammatory, antioxidant, antimicrobial, anticancer) [88], cis-α-bisabolene [34] (anti-inflammatory, antimicrobial, anticancer) [36], and γ-1-cadinene [34] (anti-inflammatory, antifungal) [37], among others. This rich phytochemical diversity positions *M. asiatica* as a promising source of bioactive compounds with therapeutic potential.

Phytochemical profiles can vary significantly between species within the *Mentha* genus. While many species in this genus are known for their essential oils rich in menthol, menthone, and carvone, *M. asiatica* Boriss. exhibits a broader spectrum of phytochemicals, particularly alkanes, alkenes, and sesquiterpenes, which are less common in other *Mentha* species. For example, compounds like dodecane [34], eicosane [34], and trans-caryophyllene [30,32,34] are more abundantly present in *M. asiatica* compared to other species such as *Mentha piperita* or *Mentha spicata*, which are typically dominated by monoterpenes like menthol. Additionally, the presence of aromatic hydrocarbons like trimethylbenzene [34] and aldehydes such as 2-hexenal [34] differentiates *M. asiatica* from its counterparts, showcasing its unique chemical composition. This variation in chemical classes suggests that *M. asiatica* may offer distinct pharmacological properties not found in other *Mentha* species, making it an intriguing candidate for further study.

The pharmacological activities of the phytochemicals found in *M. asiatica* Boriss. are diverse and promising. Alkanes such as dodecane [34] and octadecane [34] exhibit significant antimicrobial and anti-inflammatory properties, with some compounds also showing anticancer potential. Alkenes, particularly 1-eicosene [34] and 1-tetradecene [34], have demonstrated antimicrobial and antioxidant activities, while 1-octen-3-yne [34], an alkyne, is known for its antimicrobial properties [72]. Alcohols like cyclohexanol [34] and bicyclo[2.2.1]heptan-2-ol [34] add antifungal and antimicrobial activity to the plant’s pharmacological profile [73]. Aromatic hydrocarbons such as benzene [34] and trimethylbenzene [34] contribute antioxidant and antimicrobial effects, which may support the treatment of infections and oxidative stress-related conditions [75,76]. Furthermore, sesquiterpenes, including trans-caryophyllene [30,32,34] and cis-α-bisabolene [34], provide anti-inflammatory, antimicrobial, and anticancer effects [36,88]. These diverse bioactivities of the compounds in *M. asiatica* make it a valuable candidate for the development of natural therapeutic agents against a variety of health conditions, including infections, inflammation, and cancer.

The phytochemical composition of *M. asiatica* Boriss. leaves differs significantly from that of the root and stem, contributing to the variation in bioactivity across different plant parts. While the leaves are rich in alkanes, alkenes, and sesquiterpenes, the root and stem typically contain higher concentrations of essential oils and flavonoids. The leaves are particularly abundant in antimicrobial and antioxidant compounds, including dodecane [34] and 1-tetradecene [34], which are found in higher concentrations compared to the root and stem. Sesquiterpenes such as trans-caryophyllene [30,32,34] are more prevalent in the leaves, contributing to their anti-inflammatory and antimicrobial properties. On the other hand, the root might have more concentrated levels of flavonoids, which are known for their antioxidative and anti-inflammatory effects. These differences in chemical composition between the plant parts suggest that the leaves of *M. asiatica* may be more suitable for antimicrobial and antioxidant applications, while the roots and stems could be explored for their potential anti-inflammatory activities.

## 6. Antimicrobial Compounds of *M. asiatica* Boriss.

### 6.1. Alkanes, Alkenes, and Alkynes

The alkanes identified from *M. asiatica* Boriss. exhibit notable pharmacological activities, primarily antimicrobial and antioxidant properties. These compounds include undecane, dodecane, tetradecane, pentadecane, hexadecane, heptadecane, octadecane, eicosane, heneicosane, docosane, tricosane, tetracosane, pentacosane, hexacosane, heptacosane, octacosane, nonacosane, hentriacontane, dotriacontane, octatetracontane, and hexatriacontane. Such alkanes contribute to the plant’s bioactivity, making it a potential candidate for developing therapeutic agents targeting oxidative stress and microbial infections [34,41,42,43,44,45,46,47,48,49,50,51,52,53,54,55,56,57,58,59,60,61,62,63,64,65,66,67].

Among the alkanes mentioned, dodecane and octadecane have been extensively documented in scholarly sources for their pronounced antimicrobial and antioxidant capabilities. These substances exhibit notable efficacy attributed to their molecular configurations, which facilitate the disruption of microbial cell membranes and enhance their ability to scavenge free radicals. For instance, dodecane’s antimicrobial activity is primarily mediated through its ability to integrate into the lipid bilayer of microbial cell membranes, causing increased permeability and the eventual disruption of membrane integrity. Its antimicrobial activity is attributed to its ability to disrupt microbial cell membranes, leading to cell lysis and inhibition of growth. Furthermore, n-dodecane’s antiadhesion properties prevent the initial attachment of pathogens, including *Candida* species, to surfaces, which is a critical step in biofilm formation. This dual functionality is particularly effective in reducing the virulence of biofilm-associated infections. Research into the ability of yeast strains like *Rhodotorula glutinis* CMGB-RG1 to metabolize n-dodecane has further demonstrated its potential as a biosurfactant precursor, offering a natural and sustainable approach to developing antimicrobial and antiadhesion agents for medical and industrial applications [91].

For octadecane, its antimicrobial mechanism involves the destabilization of microbial cell membranes through hydrophobic interactions. This disruption compromises membrane fluidity and leads to oxidative stress in microbial cells, as ROS accumulate and damage cellular structures, such as DNA, proteins, and lipids. The dual action of membrane disruption and ROS generation positions octadecane as an effective agent against a wide range of pathogens, including biofilm-associated infections [87].

Moreover, among the alkenes and alkynes derived from *M. asiatica Boriss.*, compounds such as 1-eicosene, 1-tetradecene, 3-eicosene, 1,6,10-dodecatriene, and 1-octen-3-yne have demonstrated notable antimicrobial properties. The antimicrobial mechanisms of these compounds often involve interference with the structural integrity and functionality of microbial membranes. For instance, 1-eicosene and 1-tetradecene are known to exhibit hydrophobic interactions with lipid bilayers, leading to increased membrane fluidity and eventual leakage of intracellular contents. Additionally, alkenes like 1,6,10-dodecatriene generate ROS within microbial cells, contributing to the oxidative damage of vital cellular components. Similarly, 1-octen-3-yne disrupts membrane potential by altering proton gradients, which impairs energy production and metabolic activity in microbial cells. These mechanisms collectively underscore the potential of alkanes, alkenes, and alkynes from *Mentha asiatica Boriss.* as promising candidates for antimicrobial therapies [39,68,69,70,71].

### 6.2. Aldehydes

Aldehydes such as 2-Hexenal and 2-Decenal are known for their potent antimicrobial properties, disrupting microbial growth through mechanisms affecting cellular structures and metabolic pathways [34,77,78]. For example, 2-Hexenal has been shown to dose-dependently inhibit the mycelial growth of *Botrytis cinerea*, a necrotrophic pathogen responsible for gray mold in tomatoes, leading to pre- and post-harvest losses. Studies revealed that 2-Hexenal causes significant morphological alterations in fungal hyphae, including distortion and cytoplasmic leakage. Transcriptomic analysis indicated its impact on 3,893 differentially expressed genes, influencing pathways related to the cell wall, membrane integrity, and energy metabolism, particularly reducing ergosterol biosynthesis by downregulating key genes like *ERG1*, *ERG3*, and *ERG4*. These effects highlight its potential as a natural preservative for extending the shelf life of fruits and vegetables by effectively targeting fungal pathogens [91].

### 6.3. Phenolics

Among the aromatic compounds reported from *M. asiatica Boriss.*, thymol, carvacrol, benzene, and benzene,1,2,3-trimethyl have exhibited both antimicrobial and antioxidant properties, underscoring their therapeutic potential. Thymol and carvacrol, both phenolic compounds, exert their antimicrobial effects primarily by disrupting microbial cell membranes, whereas benzene and benzene,1,2,3-trimethyl exhibit antimicrobial activity through mechanisms involving alterations to the microbial membrane structure and inhibition of cellular respiration. These compounds integrate into the hydrophobic core of microbial membranes, destabilizing the lipid bilayer and reducing membrane integrity. This disruption hinders the proper functioning of membrane-bound proteins, including those involved in electron transport chains, thereby impairing energy production and metabolic activity in microbial cells. Additionally, the antioxidant properties of thymol and carvacrol enhance their antimicrobial efficacy by neutralizing reactive oxygen species (ROS) generated during microbial metabolism. This dual activity helps protect host cells from oxidative damage while targeting microbial pathogens. Collectively, these aromatic compounds from *M. asiatica Boriss.* represent promising candidates for developing antimicrobial agents with potential applications in medicine and industry [30,34,75,76].

### 6.4. Terpenes and Derivatives

Terpenes and their derivatives, including dl-limonene, eucalyptol, m-mentha-3(8),6-diene, α-terpinolene, and piperitenone oxide, are well-regarded for their antimicrobial properties and have demonstrated effectiveness against a variety of pathogens. dl-limonene, a monoterpene found in citrus oils, is particularly active against Gram-positive bacteria and fungi like *S. aureus*, where it disrupts microbial cell membranes, leading to the leakage of cellular contents [84]. Eucalyptol (1,8-cineole), derived from eucalyptus oil, exhibits broad-spectrum antimicrobial activity, especially against respiratory pathogens such as *S. pneumoniae* and *Haemophilus influenzae*, while also inhibiting biofilm formation and reducing bacterial virulence [85]. m-Mentha-3(8),6-diene, a menthol derivative, shows antifungal properties, particularly against *C. albicans*, by affecting fungal cell wall integrity [86]. α-terpinolene, found in pine and citrus oils, has demonstrated activity against *E. coli* and *Aspergillus niger*, working through membrane disruption and interference with microbial metabolic processes [88]. Piperitenone oxide, present in mint oils, exhibits antifungal effects, notably against *Candida* species, inhibiting hyphal growth and biofilm formation [36,88]. These terpenes offer promising alternatives to traditional antimicrobial agents, particularly in pharmaceutical and agricultural applications, due to their ability to target a wide range of bacterial, fungal, and viral pathogens [36].

### 6.5. Sesquiterpenes and Their Derivatives

Sesquiterpenes and their derivatives, including *trans*-caryophyllene, *cis*-*α*-bisabolene, *γ*-1-cadinene, *γ*-muurolene, *α*-guaiene, isolongifolene, and 9,10-dehydro-aromadendrene, exhibit significant antimicrobial properties, rendering them as promising candidates for both pharmaceutical and agricultural use [30,32,34]. These compounds demonstrate a diverse array of activities, particularly against bacterial and fungal pathogens, through mechanisms such as the disruption of microbial cell membranes, the inhibition of growth, and the modification of cellular functions [35,36,37,38,88,89,90].

For instance, the antimicrobial effect of caryophyllene was examined using human pathogenic bacterial and fungal strains, demonstrating selective antibacterial activity, particularly against *S. aureus* (with a MIC of 3 ± 1.0 µM), and more pronounced antifungal activity compared to kanamycin. The antioxidant properties of caryophyllene were assessed using DPPH and FRAP (ferric reducing antioxidant power) assays, confirming its significant antioxidant potential. Furthermore, caryophyllene exhibited anti-proliferative effects against a series of human cancer cell lines, including selective cytotoxicity against colorectal cancer cells (IC50 19 µM). The results indicated that caryophyllene induces apoptosis through mechanisms such as nuclear condensation, fragmentation, and the disruption of mitochondrial membrane potential. Additionally, it inhibited clonogenicity, migration, invasion, and spheroid formation in colon cancer cells, reinforcing its potential as an anticancer agent. These findings support the notion that caryophyllene, the active principle in *A. crassnia*, is responsible for both the selective anticancer and antimicrobial activities of the plant. As such, *β*-caryophyllene holds great promise for further development as a chemotherapeutic agent for treating colorectal malignancies [92].

Moreover, cis-*α*-Bisabolene, found in chamomile, exhibits both antifungal effects against *C. albicans* and *Aspergillus niger* and antibacterial activity against *S. aureus* and *P. aeruginosa* [93,94]. *γ*-1-Cadinene, from ginger and sandalwood, disrupts microbial cell membranes and inhibits fungal growth, particularly against *C. albicans* and *Ba. subtilis* [22]. γ-Muurolene, found in pine and cedar oils, effectively targets fungal infections, notably *Aspergillus flavus* and *C. albicans*, by compromising the fungal cell wall [95]. α-Guaiene, found in rosemary and ginger, disrupts bacterial membranes and inhibits fungal growth, proving effective against *S. aureus*, *E. coli*, and *C. albicans* [96]. Isolongifolene, found in various plant oils, has shown antibacterial activity, particularly against *P. aeruginosa* and *S. aureus*, while also preventing biofilm formation, which is crucial for managing chronic bacterial infections [97]. Lastly, 9,10-dehydro-aromadendrene exhibits broad-spectrum antimicrobial activity against both bacteria and fungi, including *C. albicans* and *E. coli*, disrupting cellular functions and making it effective against resistant pathogens [98,99]. These sesquiterpenes, with their natural origins and broad antimicrobial profiles, hold potential for use in both pharmaceutical and agricultural applications, particularly as resistance to synthetic antibiotics continues to rise.

## 7. Conclusions

These results emphasize the importance of selecting appropriate extraction methods based on the specific compounds of interest and their intended pharmacological applications. The advanced extraction methods used in this study not only provided higher yield and diversity of compounds, but also preserved the integrity of thermolabile components, thereby enhancing the therapeutic potential of Asian mint extract.

This review consolidates evidence of the antimicrobial activities of *M. asiatica* Boriss., emphasizing its bioactive compounds as promising natural alternatives to synthetic antimicrobials. Essential oils, rich in menthol, menthone, and carvone, exhibit potent bactericidal and fungicidal properties, with significant implications for combating antibiotic-resistant pathogens. Additional phytochemicals, including terpenoids, further enhance the plant’s antimicrobial efficacy. These findings reinforce the value of *M. asiatica* in traditional medicine and modern pharmacology. For instance, the aromatic compounds thymol, carvacrol, benzene, and benzene,1,2,3-trimethyl have shown potent antimicrobial effects, primarily through mechanisms involving microbial cell membrane disruption and inhibition of cellular respiration. Alkanes such as dodecane and octadecane exhibit antimicrobial properties through microbial membrane destabilization and anti-adhesion capabilities, with implications for preventing biofilm-associated infections. Additionally, terpenoids contribute to the plant’s bioactivity, further enhancing its antimicrobial and antioxidant efficacy.

The increasing prevalence of antibiotic resistance necessitates the urgent exploration of alternative antimicrobial agents. *M. asiatica* represents a compelling candidate for this purpose, offering a multifaceted phytochemical arsenal with broad-spectrum activity. However, translating its potential into practical applications requires comprehensive studies on its mechanisms of action, synergistic effects among its compounds, and interactions with other antimicrobials. Additionally, clinical evaluations are essential to confirm its safety, efficacy, and bioavailability in humans. This review also identifies knowledge gaps, such as the need for standardized methodologies in assessing antimicrobial properties and the exploration of less-studied compounds. By addressing these challenges, *M. asiatica* could contribute significantly to the development of sustainable, plant-based solutions for healthcare and food preservation, underscoring its importance as a bioresource in combating global antimicrobial resistance.

## Figures and Tables

**Table 1:** Extraction technologies and bioactive compounds from *M. asiatica Boriss.*: methods, chemical profiles, and pharmacological potentials.

Technologies for Obtaining Extracts	Extraction Parameters	Methods for Determining Biologically Active Substances	Pharmacological Activity	Chemical Classes of Main Constituents	Main Isolated Compounds	Reference
Water distillation	100 °C at atmospheric pressure, 1 atm, distillation time based on volume, condensation below 50 °C, cooling with water or refrigerant, pre-treated feed water, controlled heating rate, borosilicate glass or stainless steel apparatus, sterile collection containers, single or multiple distillation stages, optimized energy consumption	GC–MS	Antioxidant activity, antimicrobial properties	oxygenated monoterpenes, terpenoids	*trans*-piperitone oxide (64.51%), piperitenone oxide (12.34%)	[28]
SD	Condensation below 50 °C, cooling with water or refrigerant, pre-treated feed material, controlled steam flow rate, stainless steel or glass apparatus, sterile collection containers, phase separation by decantation or separatory funnel, single or multiple distillation stages	GC, GC–MS	-	monoterpenes, ketones	piperitone (67.6%), isomenthone (6.6%), *cis*-piperitol (4.2%), carvone (16.2%), pulegone (4.1%)	[29]
PTE	Purge gas (inert gas like nitrogen or helium), purge time (5 to 20 minutes), trap material (e.g., activated carbon, Tenax), trap temperature (typically −10 °C to 5 °C), desorption temperature (200 °C to 300 °C), flow rate (40–200 mL/min), sample volume (varies based on sensitivity and analyte concentration)	GC–MS	Aromatic profile characterization	terpenes, esters	linalool, linalyl acetate	[30]
Lipophilic solvent extraction	Dichloromethane, solvent volume (typically 5–10 mL for liquid samples), extraction time (ranging from 30 minutes to several hours), sample volume (varies based on sample concentration and volume), temperature (room temperature or slightly elevated), agitation (manual shaking or mechanical stirring), separation method (e.g., centrifugation or decantation), phase separation (after extraction, separate the solvent from the sample matrix)	GC–QTOF–MS, GC–FID	Antioxidant capacity (DPPH, ABTS), antibacterial effects against *S. aureus*, *C. albicans*, *B. subtilis*, *E. coli*	monoterpenes, sesquiterpenes	α-thujene, camphene, sabinene, *β*-pinene, *β*-cymene, limonene, camphol, carvone, piperitone oxide, caryophyllene oxide	[31]
Silica gel column chromatography, prep-GC	TLC-based fractionation; prep-GC for compound isolation	¹H and ¹³C NMR, GC–QTOF–MS	Antibacterial activity, ergosterol synthesis inhibition in *C. albicans*	terpenes, phenols	sabinene, limonene, *β*-caryophyllene, piperitone oxide, thymol, 4-hydroxypiperidone	[32]
SD	Condensation below 50 °C, cooling with water or refrigerant, pre-treated feed material, controlled steam flow rate, stainless steel or glass apparatus, sterile collection containers, phase separation by decantation or separatory funnel, single or multiple distillation stages	GC, GC–MS, NMR	-	monoterpenoids	rosefuran oxide (63.17%), rosefuran (11.56%)	[33]
Water distillation	100 °C at atmospheric pressure, 1 atm, distillation time based on volume, condensation below 50 °C, cooling with water or refrigerant, pre-treated feed water, controlled heating rate, borosilicate glass or stainless steel apparatus, sterile collection containers, single or multiple distillation stages, optimized energy consumption	GC–MS	Antioxidant, antimicrobial: *S. aureus*, *E. faecalis*, *E. coli*, *P. aeruginosa*, *C. albicans*	terpenes, aldehydes, alcohols, ketones, aromatic hydrocarbons, ethers, fatty acids, phenolic compounds	dl-limonene (3.05%), γ-terpinene (1.12%), trans-caryophyllene (9.51%), piperitone oxide (49.29%) 2-hexenal, 2-decenal, bicyclo[2.2.1]heptan-2-one, naphthalene, 5-methyl-2-(1-methylethyl) (1.48%)	[34]
UAE	70 g plant material soaked in 340 mL 90% ethanol for 30 min; ultrasound bath (35 W, 40 kHz) for 30 min per cycle, 2 cycles at 20 ± 1 °C; filtration and rotary evaporation	GC–MS	Antioxidant, antimicrobial: *S. aureus*, *E. faecalis*, *E. coli*, *P. aeruginosa*, *C. albicans*	sesquiterpenes, alkenes, terpene alcohols, fatty acids	piperitenone oxide (87.65%) 3-eicosene (e) (1.71%) trans-caryophyllene (0.91%) ethyl 9,12,15-octadecatrienoate (2.87%)	[34]
Vortex-assisted extraction method (VAE)	70 g plant material, 340 mL 90% ethanol; vortex mixing for 110 min at ambient temperature; filtration, rotary evaporation at ≤40°C, freeze-drying at −80°C for 12 hours	GC–MS	Antioxidant, antimicrobial: *S. aureus*, *E. faecalis*, *E. coli*, *P. aeruginosa*, *C. albicans*	alkanes, cycloalkanes	dodecane (15.92%), hexadecane (16.22%), tetradecane (14.12%)	[34]
Soxhlet apparatus method	70 g plant material; 340 mL 90% ethanol; 300 min, 5 extraction cycles in Soxhlet apparatus with boiling and condensing solvent; filtration, rotary evaporation, freeze-drying at −80 °C for 12 h.	GC–MS	Antioxidant, antimicrobial: *S. aureus*, *E. faecalis*, *E. coli*, *P. aeruginosa*, *C. albicans*	sesquiterpenes, alkenes, terpene alcohols, fatty alcohols, phenolic derivatives, cyclic structures	trans-caryophyllene (1.72%), cis-α-bisabolene (9.69%) 3-eicosene (e) (1.27%) 7-oxabicyclo[4.1.0]heptan-2-one, stigmast-5-en-3-ol (1.24%)	[34]

**Table 2:** Identified compounds from *M. asiatica* Boriss. leaves.

No	Chemical Classes	Compounds	Chemical Structure	Biological Activities
1	Alkanes	Undecane [34]	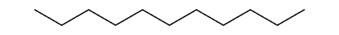	Antimicrobial [41], antifungal, antioxidant [42]
2		Dodecane [34]	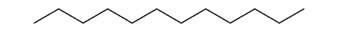	Antifungal [43], antimicrobial [44], antioxidant [45]
3		Tetradecane [34]	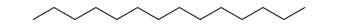	Antimicrobial [46], antifungal [47]
4		Pentadecane [34]	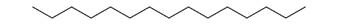	Antimicrobial and antitumor [48]
5		Hexadecane [34]	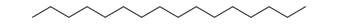	Antibacterial, antifungal, antioxidant [49]
6		Heptadecane [34]	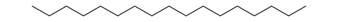	Antioxidant, cytotoxic and antimicrobial [50]
7		Octadecane [34]	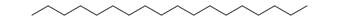	Anti-inflammatory [51], antifungal [52], antimicrobial [53]
8		Eicosane [34]	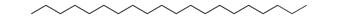	Antimicrobial and antioxidant [54]
9		Heneicosane [34]	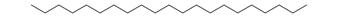	Antimicrobial and antifungal [55]
10		Docosane [34]	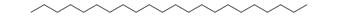	Antibacterial and antifungal [56]
11		Tricosane [34]	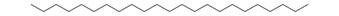	Antimicrobial [57], antioxidant [58]
12		Tetracosane [34]	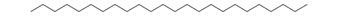	Anti-bacterial, anti-inflammatory, anti-cancer, antifungal, antioxidants [59]
13		Pentacosane [34]	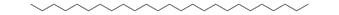	Antioxidants and anti-inflammatory [60]
14		Hexacosane [34]	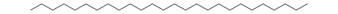	Antioxidant and antimicrobial [61]
15		Heptacosane [34]	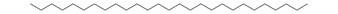	Anti-inflammatory and antioxidant [62]
16		Octacosane [34]	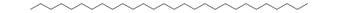	Antiinflammatory, anticancer, antifungal, antioxidants [63]
17		Nonacosane [34]	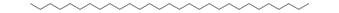	Antioxidant [64]
18		Hentriacontane [34]	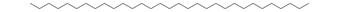	Antioxidant and antibacterial [65]
19		Dotriacontane [34]	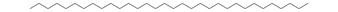	Antifungal [66]
20		Octatetracontane [34]	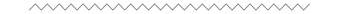	Antioxidant [67]
21		Hexatriacontane [34]	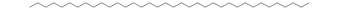	-
22		Cyclotetradecane [34]	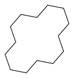	-
23		Cyclododecane [34]	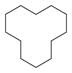	-
24		Tritetracontane [34]	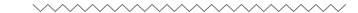	-
25	Alkenes	3-Octene, *(Z)* [32,34]	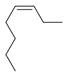	-
26		*E*-1,6-Undecadiene [34]	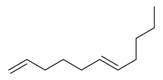	-
27		1-Ethenyl-1-isopropenylcyclohexane [34]	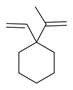	-
28		5-Methylene-1-cyclooctene [34]	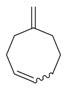	-
29		1-Butene [34]	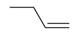	-
30		1-Undecene, 8-methyl [34]	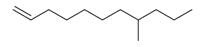	-
31		1-Eicosene [34]	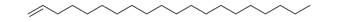	Antimicrobial, anticancerous [68]
32		1-Tetradecene [34]	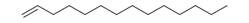	Antimicrobial and antioxidant [69]
33		3-Eicosene [34]	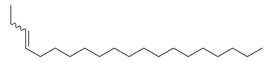	Antimicrobial and antioxidant [70]
34		4-Octene [34]		-
35		1,4,9-Decatriene [34]	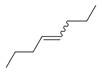	-
36		9-Eicosene [34]	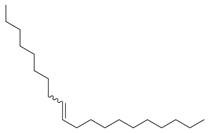	-
37		1,6,10-Dodecatriene [34]	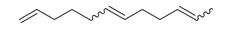	Antimicrobial [71]
38		1-Tetradecene [34]	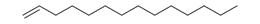	Antimicrobial, anticancerous [39]
39		3-Octene, *(Z)* [32,33,34]	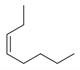	-
40		E-1,6-Undecadiene [34]	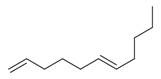	-
41		1-Ethenyl-1-isopropenylcyclohexane [34]	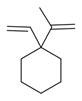	-
42		5-Methylene-1-cyclooctene [34]	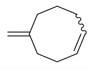	-
43	Alkynes	1-Octen-3-yne [34]	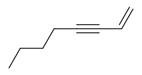	Antimicrobial [72]
44		1-Nonen-3-yne [34]	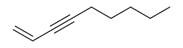	-
45	Alcohols	Cyclohexanol, 2-methyl-5-(1-methylethenyl)- [34]	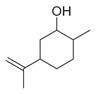	Antimicrobial, antifungal [73]
46		Hexadeca-2,6,10,14-tetraen-1-ol [34]	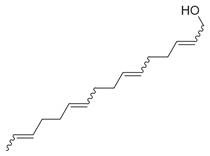	-
47		Bicyclo[2.2.1]heptan-2-ol [34]	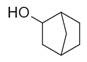	Antimicrobial, sedative [74]
48	Aromatic hydrocarbons	Benzene, 1-methyl-2-(1-methylethyl) [34]	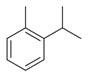	-
49		Naphthalene, decahydro-2,3-dimethoxy- [34]	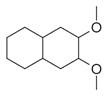	-
50		alpha-Himachalene [34]	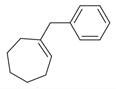	-
51		Benzene [34]	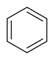	Antimicrobial, antioxidant [75]
52		Trimethylbenzene [34]	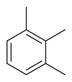	Antioxidant, antimicrobial [76]
53	Aldehydes	2-Hexenal [34]	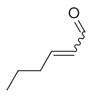	Antimicrobial [77]
54		2-Furancarboxaldehyde [34]	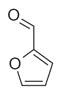	-
55		2-Decenal [34]	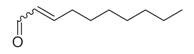	Antimicrobial [78]
56		1-Cyclohexene-1-carboxaldehyde [30,32]	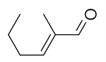	-
57		Nonanal [30,32]	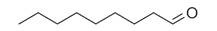	-
58		2-Decadienal [30,32]	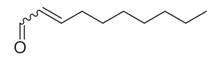	-
59	Aziridine derivatives	3-[N-Aziridylmethyl]-2-norbornanone [34]	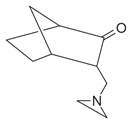	-
60	Bicyclic ketones	7-Oxabicyclo[4.1.0]heptan-2-one, 6-methyl-3-(1-methylethyl) [34]	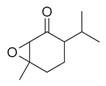	-
61	Brominated alcohols	Ethanol, 2-bromo-	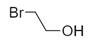	-
62	Bicyclic compounds	7-Methylene-9-oxabicyclo[6.1.0]non-2-ene [34]	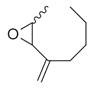	-
63		4,4-Dimethyl-3-(3-methyl-3-buten-1 -yliden)-2-methylidenbicyclo [4.1.0] heptane [34]	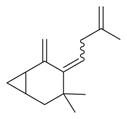	-
64		8-Oxabicyclo[5.1.0]octane [34]	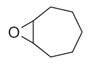	-
65	Chlorinated aldehydes	Acetaldehyde, (3-chloro-5,5-dimethyl-2-cyclohexen-1-ylidene)-, *(E)-* [34]	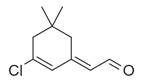	-
66	Cycloalkanes	Hexatriacontane [34]	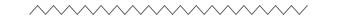	-
67		Cyclopropane [34]	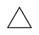	-
68		Cyclotetradecane [34]	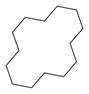	-
69		Cyclododecane [34]	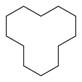	-
70	Cyclic dienes	1,5-Cyclooctadiene [34]	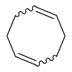	-
71		6-(1*Z*,3-Butadienyl)-1,4-cycloheptadiene [34]	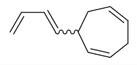	-
72	Cyclic dioxanes	2,3-Dioxabicyclo[2.2.2]oct-5-ene [34]	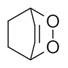	-
73	Cyclic ethers	3,8-Dioxatricyclo[5.1.0.0(2,4)]octane, 4-ethenyl- [34]	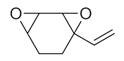	-
74	Dihydrobenzopyrans	6-Methyl-3,5,8,8a-tetrahydro-1*H*-2-benzopyran [34]	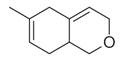	-
75	Ethers	1-Methoxy-1,4-cyclohexadiene [34]	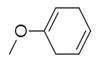	-
76		*Cis*-piperitone oxide [34]	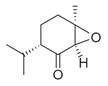	-
77		*Trans*-piperitenone oxide [34]	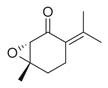	-
78		Acetic acid [30]	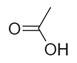	
79	Fatty acids and derivatives	Octanoic Acid [34]	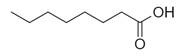	Antimicrobial [79]
80		9,12-Octadecadienoic acid (*Z,Z*)- [34]	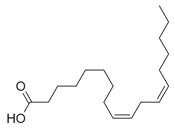	Antimicrobial [79]
81		4,7,10,13,16,19-Docosahexaenoic acid, methyl ester [34]	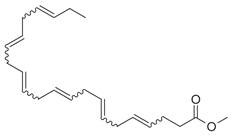	Anti-inflammatory, neuroprotective [80]
82		1-Dodecanol, 3,7,11-trimethyl- [34]	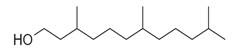	-
83	Unsaturated aldehydes	(Z)-13-Octadecenal [34]	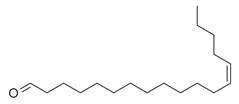	-
84	Fatty amines	Oleylamine [34]	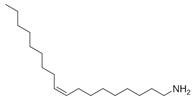	-
85	Furans	Furan, 2-(2-furanylmethyl)-5-methyl [34]	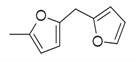	-
86	Fluorinated compounds	3-Heptafluorobutyroxydodecane [34]	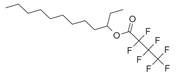	-
87	Furan derivatives	Furan-3-methanol [34]	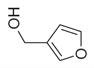	-
88	Heterocyclic compounds	3(2H)-Pyridazinone [34]	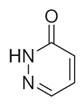	-
89	Ketones	Camphor [34]	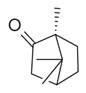	Analgesic, anti-inflammatory, antimicrobial [81]
90		Pulegone [34]	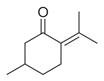	-
91		Diophenol [34]	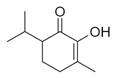	-
92		(*S*)-(+)-*cis*-Isopiperitenone [34]		Antifungal, antibacterial [82]
93		Cyclohexanone [34]	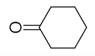	-
94		Bicyclo[3.1.1]hept-3-en-2-one [34]	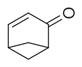	-
95		2-Cyclohexen-1-one [34]	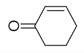	-
96	Lactones	2*H*-Pyran-2-one [34]	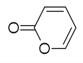	-
97	Phenolic compounds	Phenol, 2-(2-propenyl) [34]	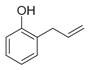	-
98		Phenol, 5-methyl-2-(1-methylethyl) [34]	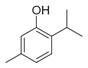	-
99		Phenol, 2-methyl-5-(1-methylethyl) [34]	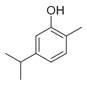	-
100		Thymol [30]	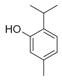	Anti-inflammatory, antimicrobial [81]
101		Carvacrol [30]	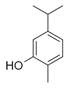	Anti-inflammatory, antimicrobial [81]
102	Sterol and derivatives	Stigmast-5-en-3-ol [30,32,34]	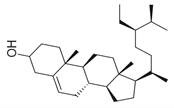	Anti-diabetic [83]
103		(22*e*)-3*β*-chloro-brassicasterol [34]	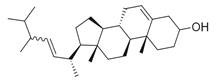	-
104	Sulfonyl chlorides	1-Octadecanesulphonyl chloride [34]	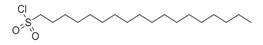	-
105	Spiro compounds	Spiro[4.5]decane, 6-methylene- [34]	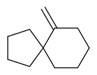	-
106		Spiro[2.2]pentane-1-carboxylic acid, 2-cyclopropyl-2-methyl- [34]	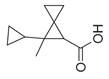	-
107	Terpenes and derivatives	*dl*-Limonene [30,32,34]	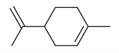	Anti-inflammatory, antioxidant, anticancer [84]
108		Eucalyptol [34]	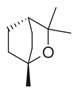	Anti-inflammatory, antioxidant, antimicrobial, anticancer [85]
109		*γ*-Terpinene [30,32,34]	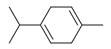	-
110		*α*-Terpinene [34]	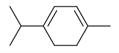	
111		*m*-Mentha-3(8),6-diene [34]	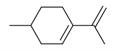	Antimicrobial, antioxidant [86]
112		*trans*-Caryophyllene [30,34]	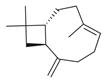	-
113		*α*-Terpinolene [30,34]	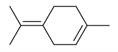	Antioxidant, sedative [87]
114		2,6-Dimethyl-1,3,5,7-octatetraene [34]	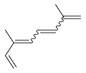	-
115		*R*(+)-Limonen [34]	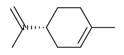	-
116		2,6-Dimethyl-1,3,5,7-octatetraene [34]	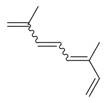	-
117		1,3,8-*p*-Menthatriene [34]	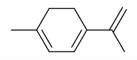	-
118		Aromadendrene [34]	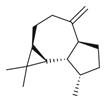	-
119		Isolongifolene, 9,10-dehydro- [34]	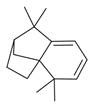	-
120		3,7,11-Trimethyl-1-dodecanol [34]	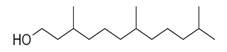	-
121		Falcarinol [30,34]	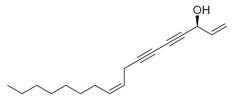	-
122		1,6,10,14-Hexadecatetraen-3-ol, 3,7,11,15-tetramethyl-, (*E,E*) [34]	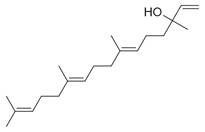	-
123		*α*-Thujene [30,32,34]	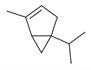	-
124		*β*-Thujene [30,32,34]	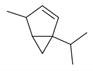	-
125		(-)-Sabinene [30,32,34]	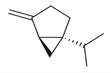	-
126		*β*-Myrcene [30]	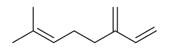	-
127		Camphene [30,32]	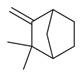	-
128		*o*-Cymene [30]	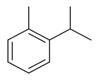	-
129		*α*-Terpinolene [30]	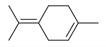	-
130		(*E*)- linalool oxide [30]	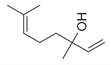	-
131		*p*-Cymen-8-ol [30]	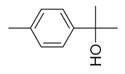	-
132		Ascaridole [30]	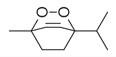	-
133		Piperitenone oxide [30,32,34]	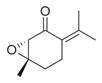	Antimicrobial activity, anti-inflammatory properties, antioxidant effects, analgesic effects, relaxant properties, insecticidal activity, gastroprotective effects [36,88]
134		6-Methyl-3,5,8,8a-tetrahydro-1*H*-2-benzopyran [34]	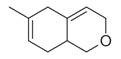	-
135		*d*-Carvone [30,34]	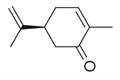	-
136	Sesquiterpenes and derivatives	*trans*-Caryophyllene [30,32,34]	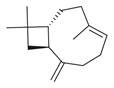	Anti-inflammatory, antioxidant, antimicrobial, anticancer [88]
137		*cis-α*-Bisabolene [34]	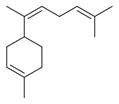	Anti-inflammatory, antimicrobial, anticancer [36]
138		*γ-1*-Cadinene [34]	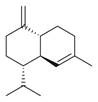	Anti-inflammatory, antifungal [37]
139		*cis-δ*-1,6,8-Iridadiene [34]	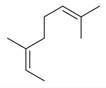	-
140		*γ*-Muurolene [34]	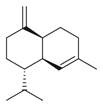	Anti-inflammatory, antimicrobial [38]
141		*α*-Guaiene [34]	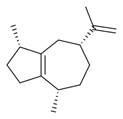	Anti-inflammatory, antimicrobial [35]
142		Isolongifolene, 9,10-dehydro- [34]	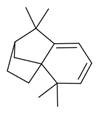	Anti-inflammatory, antioxidant [89]
143		Aromadendrene, dehydro- [34]	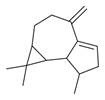	Anti-inflammatory, antimicrobial [90]
144		*α*-Amorphene [34]	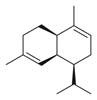	-
145		*α*-Humulene [30]	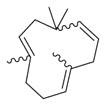	-
146		Bicyclogermacrene [30]	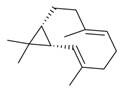	-
147		Caryophyllene oxide [30]	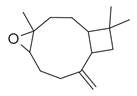	-
148		*β*-Farnesene [30]	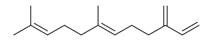	-
149	Terpenoids	Sabinene hydrate [30]	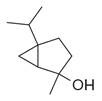	-
150	Terpenoid esters	Linalyl acetate [30]	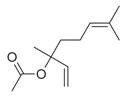	-
151		α-Fenchyl acetate [30]	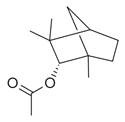	-
152	Terpenoid alcohols	4-Terpineol [30]	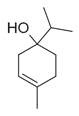	-
153		*α*-Terpineol [30]	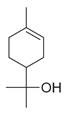	-
154		(*E*)-Piperitol [30]	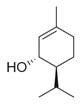	-
155		(*Z*)-Piperitol [30]	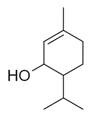	-
156	Thiepenes	1*H*-Thiepine [34]	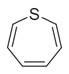	-
157	Thienopyridines	4-Methylthieno[2,3-b]pyridine [34]	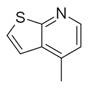	-
158	Thiophenes	(1’-butenyl)thiophene 1,1’-Bicyclopentyl [34]	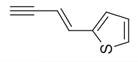	-
159	Organosilicon compounds	Docosyltrichlorosilane [34]	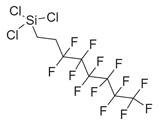	-
160	Pyrazolone derivatives	3*H*-Pyrazol-3-one, 2,4-dihydro-4,4,5-trimethyl- [34]	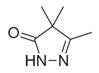	-
161	Phenylpropanoid	Anethole [30,32,34]	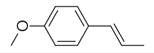	-

## Data Availability

Not applicable.
